# TRENDS IN CHRONIC DISEASE SPENDING AND HEALTH OUTCOMES AMONG MEDICARE FFS BENEFICIARIES IN PUERTO RICO

**DOI:** 10.1093/geroni/igae098.3913

**Published:** 2024-12-31

**Authors:** Matthew Campos, Maricruz Rivera-Hernandez

**Affiliations:** Brown University School of Public Health, Providence, Rhode Island, United States; Brown University School of Public Health, Providence, Rhode Island, United States

## Abstract

There is growing recognition of disparities in chronic disease outcomes between Puerto Ricans on the island and those on the US mainland, yet comparative data are limited. We assessed the prevalence of key chronic conditions—Alzheimer’s disease and related dementias (ADRD), diabetes, and chronic kidney disease (CKD)—and total care costs for Medicare fee-for-service (FFS) beneficiaries in Puerto Rico (PR) compared to states with high older adult (65+) Puerto Rican populations [Florida (FL), New York (NY), Georgia (GA), New Jersey (NJ), California (CA)] from 2015 – 2020. We used data from the Mapping Medicare Disparities Tool from the Center for Medicare and Medicaid Services. Outcomes of interest included age-adjusted prevalence and risk-adjusted total costs per beneficiary. From 2015 – 2020, age-adjusted prevalence trends in PR showed decreases in ADRD by three percentage points (13% to 10%) and diabetes by six percentage points (46% to 40%). Conversely, CKD prevalence increased by 12 percentage points from 21% to 33%. Despite ADRD trends aligning with national averages, diabetes (PR: 40%, Nation: 26%, FL: 28%, NY: 27%, GA: 29%, NJ: 29%, CA: 24%) and CKD (PR: 33%, Nation: 25%, FL: 29%, NY: 24%, GA: 30%, NJ: 25%, CA: 23%) rates in Puerto Rico remain notably higher. Additionally, PR risk-adjusted average total spending per beneficiary in 2020 was below national averages for all assessed chronic conditions (ADRD: $13,037 vs. $20,678; diabetes: $11,363 vs. $15,525; CKD: $13,181 vs. $18,376). These disparities suggest potential impacts on access and quality of care and highlight the need for policy interventions.

